# Numerical Simulation Study on the Elastic Behaviour of Single-Walled Boron–Group 15 Nanotubes: A Comparative Assessment

**DOI:** 10.3390/ma19214483

**Published:** 2026-10-24

**Authors:** Nataliya A. Sakharova, Eduardo B. Matos, André F. G. Pereira, José V. Fernandes, Jorge M. Antunes

**Affiliations:** 1 UnIRE, Instituto Superior de Engenharia de Lisboa, Universidade Politécnica de Lisboa, Rua Conselheiro Emídio Navarro 1, 1959-007 Lisboa, Portugal; 2 Centre for Mechanical Engineering, Materials and Processes (CEMMPRE), Advanced Production and Intelligent Systems, Associated Laboratory (ARISE), Department of Mechanical Engineering, University of Coimbra, Rua Luís Reis Santos, Pinhal de Marrocos, 3030-788 Coimbra, Portugal; andre.pereira@dem.uc.pt (A.F.G.P.); valdemar.fernandes@dem.uc.pt (J.V.F.); jorge.antunes@dem.uc.pt (J.M.A.); 3 Process Metallurgy Research Unit, University of Oulu, P.O. Box 4300, 90014 Oulu, Finland; eduardo.borgesmatos@oulu.fi; 4 Abrantes High School of Technology, Polytechnic Institute of Tomar, Quinta do Contador, Estrada da Serra, 2300-313 Tomar, Portugal

**Keywords:** group 15 element, boron nanotubes, bond length, force field constants, rigidity, elastic moduli, modelling, numerical simulation

## Abstract

**Highlights:**

- A systematic comparative study of the elastic behaviour of boron–group 15 nanotubes is presented.
- Elastic properties of scarcely investigated boron arsenide and boron antimonide nanotubes are reported.
- Analytical expressions are proposed to describe the elastic response of boron–group 15 nanotubes.
- Boron–group 15 and group 13–nitride nanotubes show distinct elastic response trends.
- Equivalent beam characteristics help explain differences in the elastic response of the two nanotube families.

**Abstract:**

Non-carbon nanotubes are attractive nanostructures with potential applications in advanced electronic and high-temperature devices. Among boron–group 15 nanotubes, boron nitride nanotubes (BNNTs) have been extensively investigated, whereas the mechanical behaviour of boron phosphide (BPNTs), boron arsenide (BAsNTs) and boron antimonide (BSbNTs) nanotubes remains considerably less explored, particularly for the latter two compounds. A systematic comparative investigation of linear elastic behaviour was carried out for single-walled BNNTs, BPNTs, BAsNTs and BSbNTs over a wide range of diameters using the nanoscale continuum modelling/molecular structural mechanics (NCM/MSM) approach. Tensile, bending and torsional rigidities, surface Young’s and shear moduli, and Poisson’s ratio were assessed, and analytical expressions for their evaluation were proposed. The results were discussed in terms of nanotube diameter, chirality and equilibrium bond length, with particular attention to the influence of the group 15 atoms. Comparisons with available literature data and the previously investigated group 13–nitride nanotube family were used to identify common and distinct trends. Within the adopted modelling framework, the results provide a consistent description of elastic behaviour across the entire boron–group 15 nanotube family and reveal comparative trends that can guide further theoretical and computational studies.

## 1. Introduction

The significant advances in nanotechnology have stimulated intensive research on new low-dimensional materials with attractive physical properties for nanoscale devices. Since the discovery of graphene, numerous two-dimensional (2D) materials have emerged as promising candidates for next-generation technologies, among which boron compounds with elements of group 15 of the periodic table have received increasing research attention. This family comprises boron nitride (BN), boron phosphide (BP), boron arsenide (BAs) and boron antimonide (BSb), which exhibit attractive combinations of thermal and chemical stability, favourable thermal transport characteristics and suitable electronic properties for applications in nanoelectronics, optoelectronics, photovoltaics and devices operating under harsh conditions. Among these compounds, boron nitride has been extensively investigated over recent decades (see, for example [1,2,3,4,5,6]) and is now regarded as one of the most mature and well-explored non-carbon nanomaterials. Although boron phosphide has received considerably less attention, interest in this material has increased in recent years owing to its promising electronic and optical properties, as well as its remarkable chemical stability [7]. In contrast, boron arsenide and especially boron antimonide have only recently entered the focus of research, driven by their remarkable physical properties and potential for advanced electronic applications, with BAs attracting particular attention due to its exceptionally high thermal conductivity [8,9,10,11], and BSb because of its high carrier mobility [12] and tunability of its optical characteristics by induced strains [13].

One-dimensional (1D) nanostructures, namely nanotubes (NTs), obtained by rolling these 2D monolayers with a graphene-like structure, represent another promising class of low-dimensional materials. The transition from two-dimensional nanosheets to tubular geometries may provide additional opportunities for tailoring their physical properties and give rise to new functionalities, opening new perspectives for advanced nanoscale applications [14]. Among the boron–group 15 compounds, boron nitride nanotubes (BNNTs) were theoretically predicted and subsequently synthesized, becoming one of the most extensively investigated non-carbon nanotubes [15,16,17,18,19,20,21]. Boron phosphide nanotubes (BPNTs) were also theoretically proposed [22,23] and have recently attracted growing research interest, although studies devoted specifically to these nanotubes remain rather limited [24]. In contrast, boron arsenide (BAsNTs) and boron antimonide (BSbNTs) nanotubes remain largely unexplored and, to the best of our knowledge, are still limited to theoretical predictions, mainly focused on their electronic properties and stability [12,25,26]. Nevertheless, the growing interest in the corresponding two-dimensional compounds suggests that their one-dimensional counterparts are likely to receive increasing attention in the near future, particularly in the search for multifunctional nanomaterials for advanced technological applications, where the mechanical behaviour of the constituent nanostructures plays a fundamental role. Recent theoretical studies have highlighted the potential of BAsNTs for applications such as gas sensors [26]. In addition, the promising electronic properties reported for BAs-based two-dimensional structures and heterostructures [8,25,27,28] suggest that related nanotubular structures may also be of interest for the development of advanced electronic systems.

The successful design and reliable operation of NT-based nanodevices require a thorough understanding of the mechanical behaviour of their constituent nanostructures. Besides their attractive physical properties, the mechanical stability of nanotubes plays a key role in determining their reliability, structural integrity and long-term functionality under operating conditions. Furthermore, the possibility of tailoring the functional properties of low-dimensional materials through mechanical deformation has opened new perspectives in strain engineering, reinforcing the importance of accurately characterizing their elastic properties, including Young’s and shear moduli, as well as Poisson’s ratio. Therefore, the evaluation of these properties is essential for assessing the suitability of boron–group 15 nanotubes for future technological applications and for supporting the design and optimization of nanotube-based devices and systems.

The mechanical behaviour of boron–group 15 NTs has been investigated by means of experimental and theoretical approaches. However, the available knowledge about this family of nanomaterials remains very uneven. Among them, BNNTs have received by far the greatest research attention, and their elastic properties have been extensively characterized [18,21,22,23,24,25,26,27,28,29,30,31,32]. In contrast, studies devoted to BPNTs remain relatively limited [33,34], whereas investigations on BAsNTs and especially BSbNTs are still scarce or non-existent. To the best of our knowledge, no further studies specifically addressing the elastic properties of BPNTs have been reported since the publication of our 2022 work [34]. Moreover, available studies generally focus on individual nanotubes, making direct comparisons between different members of the boron–group 15 family difficult. Consequently, a systematic comparative investigation of the elastic properties of boron–group 15 nanotubes is still lacking.

To address the above-mentioned research gap, a systematic comparative investigation of the elastic properties of single-walled boron nitride (SWBNNTs), boron phosphide (SWBPNTs), boron arsenide (SWBAsNTs) and boron antimonide (SWBSbNTs) nanotubes is presented. The study was performed using the nanoscale continuum modelling/molecular structural mechanics (NCM/MSM) approach, considering NTs covering a wide range of chiral indices and diameters. Since the wall thickness of boron–group 15 nanotubes, except for SWBNNTs, has not yet been unambiguously established, the elastic response is characterized in terms of surface elastic moduli, thereby avoiding the use of an arbitrary wall thickness. The tensile, bending and torsional rigidities obtained from the numerical simulations were subsequently used to determine the corresponding elastic properties, including surface Young’s and shear moduli, as well as Poisson’s ratio. The present comparative study is expected to contribute to a better understanding of the mechanical behaviour of boron–group 15 nanotubes, providing a useful basis for their consideration in the development of advanced nanostructures.

## 2. Materials and Methods

### 2.1. Atomic Structure and Geometrical Characteristics of Boron–Group 15 Nanotubes

The boron–group 15 nanotubes (BNNTs, BPNTs, BAsNTs and BSbNTs) investigated in this work share a common structural arrangement derived from their corresponding two-dimensional monolayers. These monolayers consist of a hexagonal honeycomb lattice formed by alternating boron (B) and group 15 atoms (N, P, As or Sb) and, for all compounds considered in the present study, exhibit a planar geometry [35]. The atomic arrangement of these monolayers is described by the chiral vector, ***C_h_***, and the chiral angle, *θ*, expressed by Equations (1) and (2), respectively:

***C_h_*** = *n**a***_1_ + *m**a***_2_, (1)

(2) $$\theta =\sin^{-1}\frac{\sqrt{3}}{2}\frac{m}{\sqrt{\left(n^{2}+\mathrm{nm}+m^{2}\right)}},$$

where *n* and *m* are the chiral indices, *a*_1_ and *a*_2_ are the unit vectors of the hexagonal lattice, and *a* = $\sqrt{3}a_{\mathrm{B-A}15}$, where *a*_B-A15_ denotes the equilibrium bond length between boron and the corresponding group 15 atoms.

Single-walled nanotubes are generated by rolling the corresponding monolayer according to a specified chiral vector, resulting in a nanotube whose diameter (*D*_n_) is determined as follows:

(3) $$D_{n}=\frac{a_{\mathrm{B-A}15}\sqrt{3\left(n^{2}+\mathrm{nm}+m^{2}\right)}}{\pi },$$

where *a*_B-A15_ represents the equilibrium bond length of the corresponding boron–group 15 compound. The nanotubes considered in the present study have a circular cross-section.

**Figure 1:**
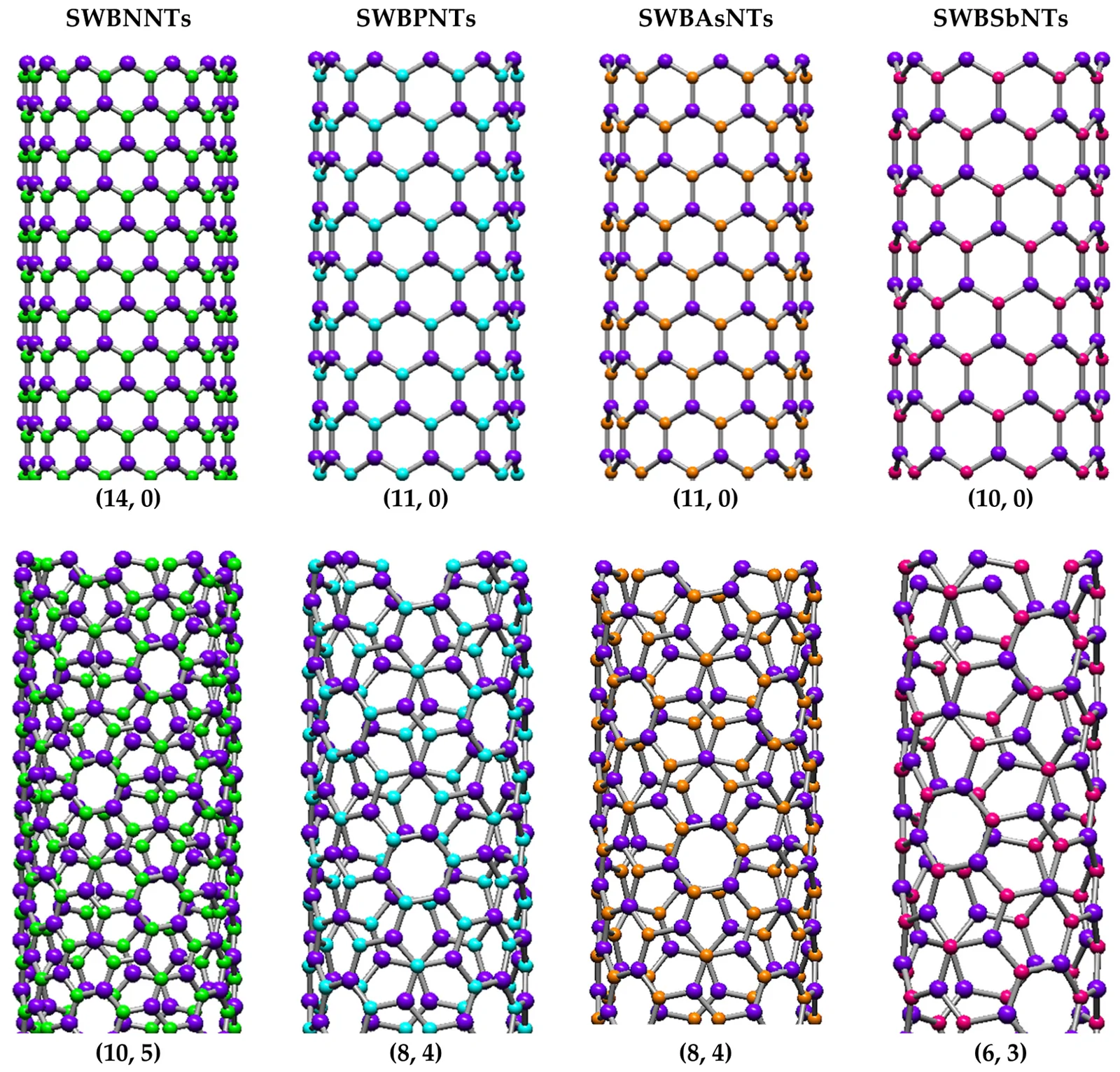

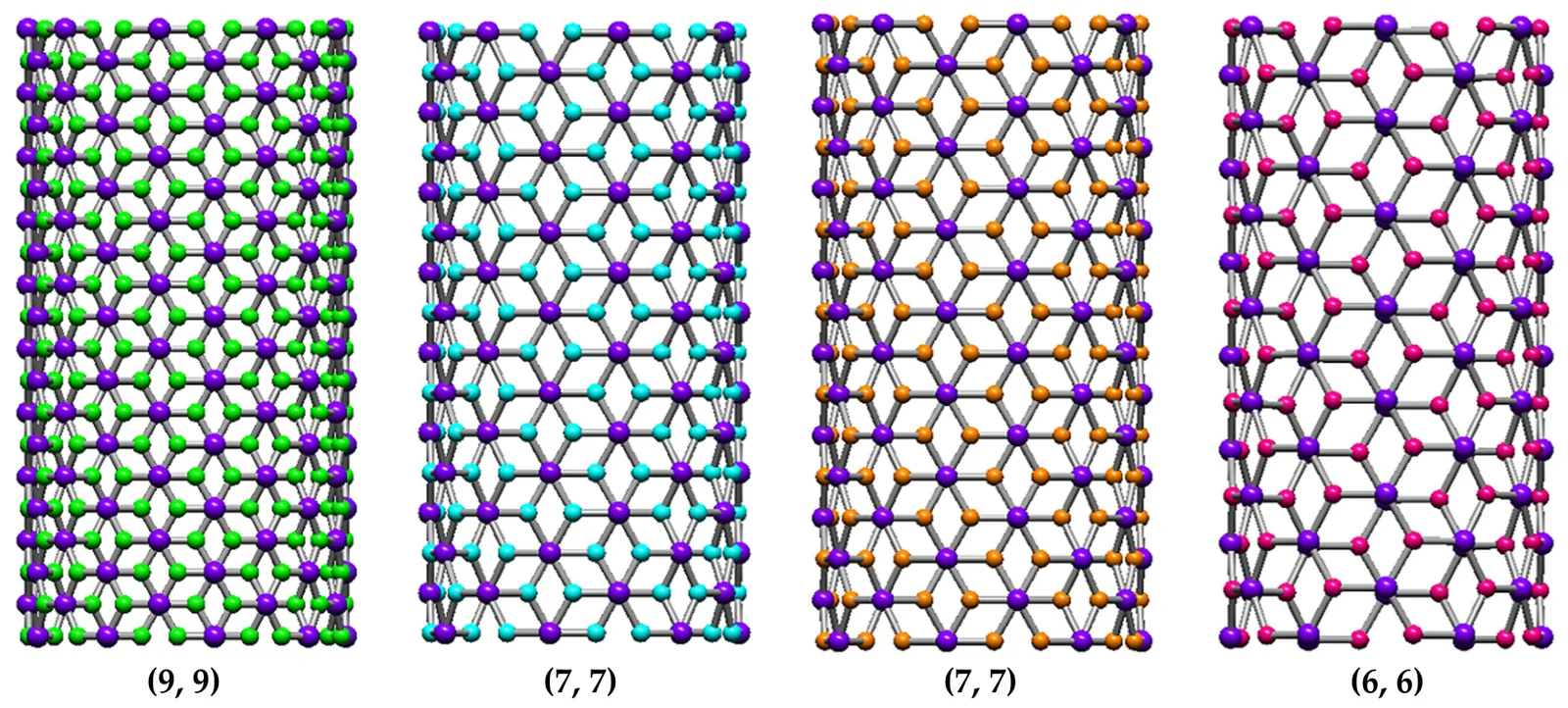
Representative structures of zigzag, chiral and armchair SWBNNTs, SWBPNTs, SWBAsNTs and SWBSbNTs, generated using Nanotube Modeler© software (version 1.8.0, ©JCrystalSoft, http://www.jcrystal.com). Boron atoms are shown in purple, nitrogen in green, phosphorus in bright blue, arsenic in orange and antimony in bright pink.

According to the chiral angle, three nanotube configurations are distinguished: zigzag (*n*, 0), chiral (*n*, *m*) and armchair (*n*, *n*), corresponding to *θ* = 0°, 0° < *θ* < 30° and *θ* = 30°, respectively. Representative structures of the three configurations considered in the present work are shown in Figure 1.

Based on the geometrical description presented above, boron–group 15 nanotubes covering the three principal configurations and a range of comparable diameters were considered in the present study. For each compound, the chiral indices were selected to provide comparable nanotube diameters, enabling a consistent assessment of their mechanical response. Nanotube lengths corresponding to approximately 30 times the respective diameters were adopted to avoid possible length effects on the calculated elastic properties [21]. The corresponding geometrical characteristics are summarized in Table 1. Zigzag and armchair nanotubes, corresponding to the two non-chiral families, together with the most populated chiral family in the investigated set, characterized by *θ* = 19.1°, were considered.

### 2.2. Equivalent Beam Modelling and Evaluation of Elastic Properties

The elastic behaviour of the boron–group 15 nanotubes was modelled using the NCM/MSM approach, in which each diatomic bond is represented by an equivalent beam element. Based on the energy equivalence between the molecular bond and the beam element, the bond stretching (*k*_r_), bond bending (*k*_θ_) and torsional resistance (*k*_τ_) force constants are related to the beam tensile (*E*_b_*A*_b_), bending (*E*_b_I_b_), and torsional (*G*_b_*J*_b_), rigidities according to the expressions [36]:

*E*_b_*A*_b_ = *lk*_r_, *E*_b_*I*_b_ = *lk*_θ_, *G*_b_*J*_b_ = *lk*_τ_, (4)

where *A*_b_ = π*d*^2^/4, *I*_b_ = π*d*^4^/64 and *J*_b_ = π*d*^4^/32 are the circular beam cross-sectional area, the moment of inertia and the polar moment of inertia, respectively, *d* is the equivalent beam diameter, and *l* is equal to the equilibrium bond length, *a*_B-A15_.

The bond stretching and bond bending force constants were derived from the surface Young’s modulus (*E*_s_), Poisson’s ratio (*ν*) and equilibrium bond length (*a*_B-A15_) according to the following equations [37]:

(5) $$k_{r}=\frac{3E_{s}}{\sqrt{3}\left(1-\nu \right)},$$

(6) $$k_{\theta }=\frac{E_{s}a_{\mathrm{B-A}15}^{2}}{2\sqrt{3}\left(1+3\nu \right)}.$$

The bond length (*a*_B-A15_), surface Young’s modulus (*E*_s_), Poisson’s ratio (*ν*), and the corresponding force constants (*k*_r_, *k*_θ_ and *k*_τ_) adopted for the boron–group 15 compounds investigated in the present study are summarized in Table 2. For BP, BAs and BSb nanostructures, *k*_τ_ was assumed from the DREIDING force field formulation [38], in which the torsional contribution is parameterized according to the hybridization of the bonded atoms. This procedure gives *k*_τ_ = 0.625 nN·nm/rad^2^ for all three compounds.

Based on these parameters, the geometrical and elastic properties of the equivalent beam elements, shown in Table 3, were obtained and used as input parameters for the finite element (FE) simulations.

The finite element meshes of the single-walled boron–group 15 nanotubes were generated using Nanotube Modeler© software (version 1.8.0, ©JCrystalSoft, http://www.jcrystal.com). The generated structural files were subsequently converted into the format required by the ABAQUS^®^ (Abaqus 2020, Dassault Systèmes^®^, Providence, RI, USA) software using the in-house InterfaceNanotubes.NM programme [21].

The finite element simulations were carried out using the ABAQUS^®^ software package. The diatomic bonds of the boron–group 15 nanotubes were replaced by equivalent two-node cubic beam elements with a circular cross-section (see Table 3). To evaluate the elastic response, three independent numerical tests corresponding to tension, bending and torsion were performed. Consequently, tensile (*EA*), bending (*EI*) and torsion (*GJ*) rigidities were evaluated according to:

(7) $$\mathrm{EA}=\frac{F_{z}L_{n}}{u_{z}},$$

(8) $$\mathrm{EI}=\frac{F_{y}L_{n}^{3}}{{3u}_{y}},$$

(9) $$\mathrm{GJ}=\frac{TL_{n}}{\varphi },$$

where *F*_z_, *F*_y_ and *T* denote the axial force, transverse force and torsional moment, respectively, applied at one end of a nanotube of length *L*_n_; *u*_z_, *u*_y_ and *φ* are the corresponding axial displacement, transverse displacement and twist angle obtained directly from the finite element analysis. At the opposite nanotube end, all degrees of freedom of the edge nodes were constrained. For the torsion test, an additional boundary condition was imposed by restraining the radial displacement of the edge nodes. The boundary and loading conditions adopted in the three numerical tests are illustrated in Figure S1 of the Supplementary Materials.

The Young’s modulus (*E*), shear modulus (*G*) and Poisson’s ratio (*ν*) were estimated from the calculated rigidities using the following expressions [21]:

(10) $$E=\frac{\mathrm{EA}}{\pi t_{n}\sqrt{8\left(\frac{\mathrm{EI}}{\mathrm{EA}}\right)-t_{n}^{2}}},$$

(11) $$G=\frac{\mathrm{GJ}}{2\pi t_{n}\left(\frac{\mathrm{EI}}{\mathrm{EA}}\right)\sqrt{8\left(\frac{\mathrm{EI}}{\mathrm{EA}}\right)-t_{n}^{2}}},$$

(12) $$\nu =\frac{E}{2G}-1=\frac{\mathrm{EI}}{\mathrm{GJ}}-1,$$

where *t*_n_ is the nanotube wall thickness.

Since these expressions depend on the nanotube wall thickness (*t*_n_), whose value has not yet been unambiguously established for most boron–group 15 nanotubes, the present work focuses on the surface elastic moduli, which are independent of wall thickness. The surface elastic moduli represent the corresponding two-dimensional elastic stiffnesses and are related to the conventional three-dimensional moduli through the adopted wall thickness, i.e., *E*_s_ = *Et*_n_ and *G*_s_ = *Gt*_n_ [40,41]. This representation is particularly convenient for single-walled nanotubes, for which the definition of an effective thickness is not unique. Assuming the thin-wall condition, $t_{n}^{2}\ll 8\left(\mathrm{EI}/\mathrm{EA}\right)$, Equations (10) and (11) become:

(13) $$E_{s}=Et_{n}=\frac{\mathrm{EA}}{\pi \sqrt{8\left(\frac{\mathrm{EI}}{\mathrm{EA}}\right)}},$$

(14) $$G_{s}=Gt_{n}=\frac{\mathrm{GJ}}{2\pi \left(\frac{\mathrm{EI}}{\mathrm{EA}}\right)\sqrt{8\left(\frac{\mathrm{EI}}{\mathrm{EA}}\right)}},$$

where *E*_s_ and *G*_s_ denote the surface Young’s and surface shear moduli, respectively.

## 3. Results and Discussion: Elastic Properties of Single-Walled Boron–Group 15 Nanotubes

### 3.1. Trends in Tensile, Bending and Torsional Rigidities

The tensile, bending and torsional rigidities of boron–group 15 nanotubes were evaluated using Equations (7)–(9). The corresponding numerical values for all investigated nanotube configurations are provided in Table S1 of the Supplementary Materials. The results revealed a consistent dependence of these rigidities on the nanotube diameter (*D*_n_), regardless of nanotube chirality and the boron–group 15 compound considered. As the nanotube diameter increases, all three rigidities increase, with the tensile rigidity exhibiting an approximately linear dependence on the nanotube diameter, whereas the bending and torsional rigidities are proportional to the cube of the *D*_n_. For a given nanotube diameter, the three rigidities exhibit a gradual decrease from SWBNNTs to SWBSbNTs, indicating lower tensile, bending and torsional rigidities with increasing equilibrium bond length.

**Figure 2:**
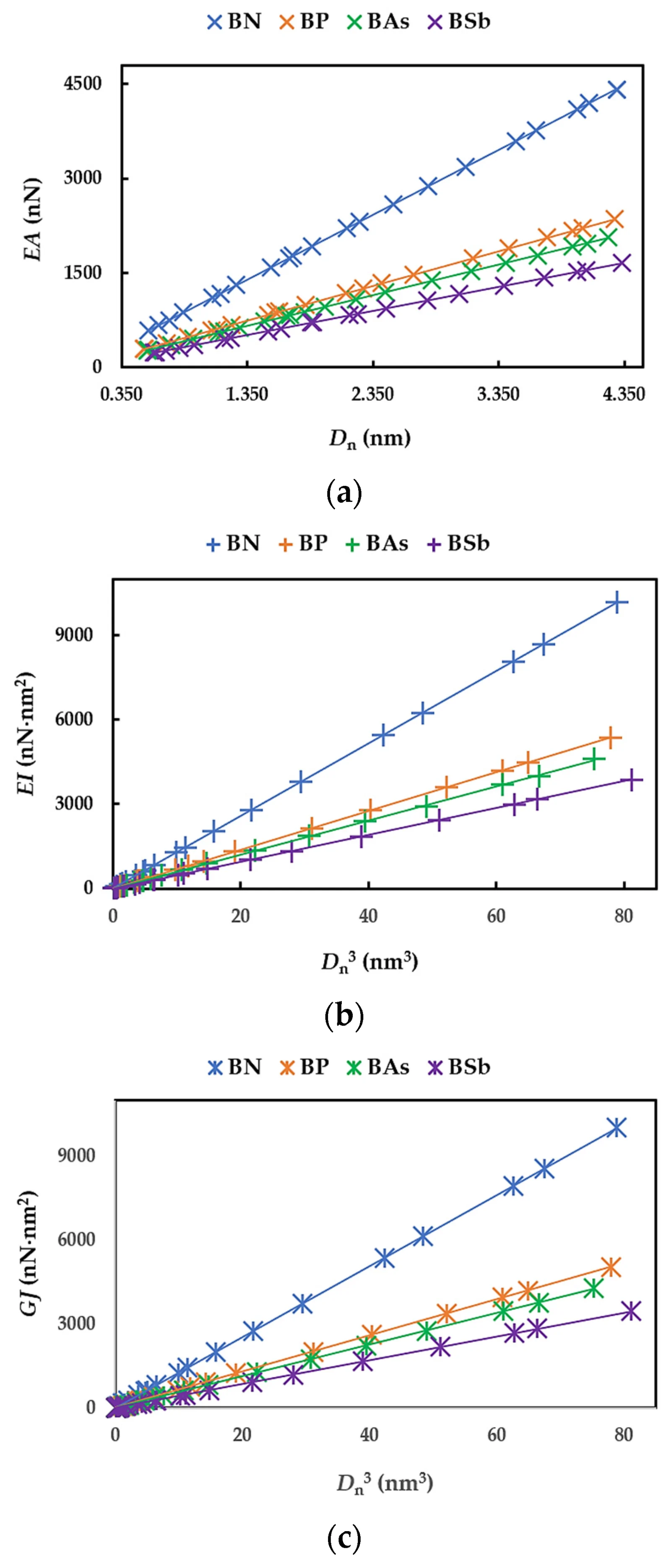
Linear fittings of: (**a**) tensile (*EA*) rigidity as a function of the NT diameter (*D*_n_), (**b**) bending (*EI*) and (**c**) torsional (*GJ*) rigidities as a function of $D_{n}^{3}$, for the single-walled BN, BP, BAs and BSb nanotubes from Table 1.

The observed trends can be accurately described by simple analytical expressions. Consistent with the behaviour previously reported for nitride [42] and phosphide [34] nanotube families, the tensile rigidity varies linearly with the nanotube diameter (*D*_n_), while the bending and torsional rigidities are proportional to the cube of the diameter, as illustrated by the linear fittings presented in Figure 2:

*EA* = *α*_B-A15_*D*_n_, (15)

(16) $$\mathrm{EI}=\beta_{\mathrm{B-A}15}{D_{n}}^{3},$$

(17) $$\mathrm{GJ}=\gamma_{\mathrm{B-A}15}{D_{n}}^{3},$$

where *α*_B-A15_, *β*_B-A15_ and *γ*_B-A15_ are fitting parameters corresponding to the tensile, bending and torsional rigidities, respectively.

The fitting parameters were obtained from the slopes of the linear regressions shown in Figure 2. In all cases, the coefficient of determination exceeded 0.9999. The fitting parameters, together with the mean relative difference between the analytical and numerical rigidities, are summarized in Table 4.

The analytical expressions reproduce the finite element rigidities with excellent accuracy, the mean relative difference remaining below 0.5%, 0.9% and 0.7% for *EA*, *EI* and *GJ* rigidities, respectively, for all nanotube compounds. The high *R*^2^ values and the small differences between the fitted and numerical results indicate that Equations (15)–(17) accurately reproduce the FE results over the investigated diameter range. Consequently, these expressions can be used to evaluate the tensile, bending and torsional rigidities of boron–group 15 nanotubes within this range.

**Figure 3:**
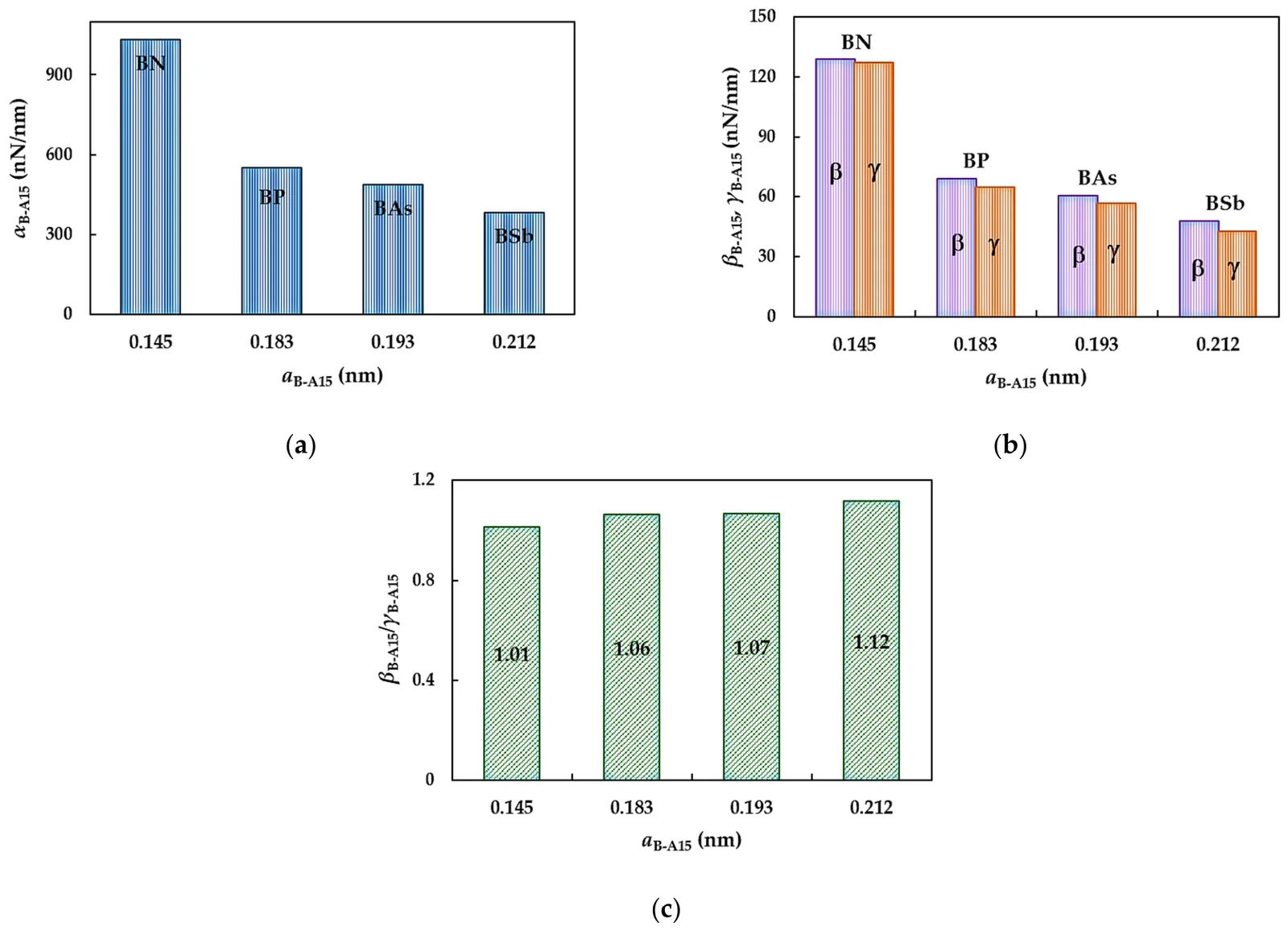
Fitting parameters as a function of the bond lengths, *a*_B-A15_: (**a**) *α*_B-A15_; (**b**) *β*_B-A15_ together with *γ*_B-A15_, and (**c**) *β*_B-A15_/*γ*_B-A15_ ratio for boron–group 15 NTs under study.

The fitting parameters also provide additional insight into the influence of compound type on the mechanical response of boron–group 15 nanotubes. As shown in Figure 3a,b, the values of *α*_B-A15_, *β*_B-A15_ and *γ*_B-A15_ decrease gradually with increasing bond length (*a*_B-A15_), reflecting the progressive reduction in tensile, bending and torsional rigidities from BNNTs to BSbNTs.

Additional insight into the relationship between the bending and torsional responses can be obtained from the ratio between the fitting parameters *β*_B-A15_ and *γ*_B-A15_, presented in Figure 3c. Since these parameters describe the bending and torsional rigidities, respectively, their ratio reflects the relative magnitude of both deformation modes. For the boron–group 15 nanotubes investigated, the *β_B-A15_*/*γ*_B-A15_ ratio increases slightly with increasing equilibrium bond length, from approximately 1.01 for BNNTs to 1.12 for BSbNTs. This behaviour indicates that the torsional rigidity decreases slightly more rapidly than the bending rigidity as the equilibrium bond length increases. In contrast, this effect is considerably less pronounced than that previously reported for group 13–nitride nanotubes [42], where the corresponding *β*/*γ* ratio increased from approximately 1.0 for boron nitride to about 1.5 for thallium nitride (TlN) NTs. Consequently, although increasing the equilibrium bond length leads to the same general tendency in both nanotube families, its influence on the relative bending and torsional responses is substantially weaker for boron–group 15 nanotubes, revealing a different influence of the group 15 atoms on the balance between bending and torsional responses.

### 3.2. Surface Young’s Modulus of Boron–Group 15 Nanotubes

The surface Young’s modulus (*E*_s_) of the boron–group 15 nanotubes was evaluated using Equation (13), which combines the tensile and bending rigidities obtained from the finite element analyses. Since these rigidities are accurately described by the analytical expressions derived in the previous section, a diameter-independent expression for the surface Young’s modulus can be readily derived by combining Equations (13), (15) and (16):

(18) $$E_{s}=\frac{\alpha_{\mathrm{B-A}15}}{\pi \sqrt{8\left(\frac{\beta_{\mathrm{B-A}15}}{\alpha_{\mathrm{B-A}15}}\right)}},$$

where *α*_B-A15_ and *β*_B-A15_ are the fitting parameters from Table 4 corresponding to the tensile and bending rigidities, respectively.

**Figure 4:**
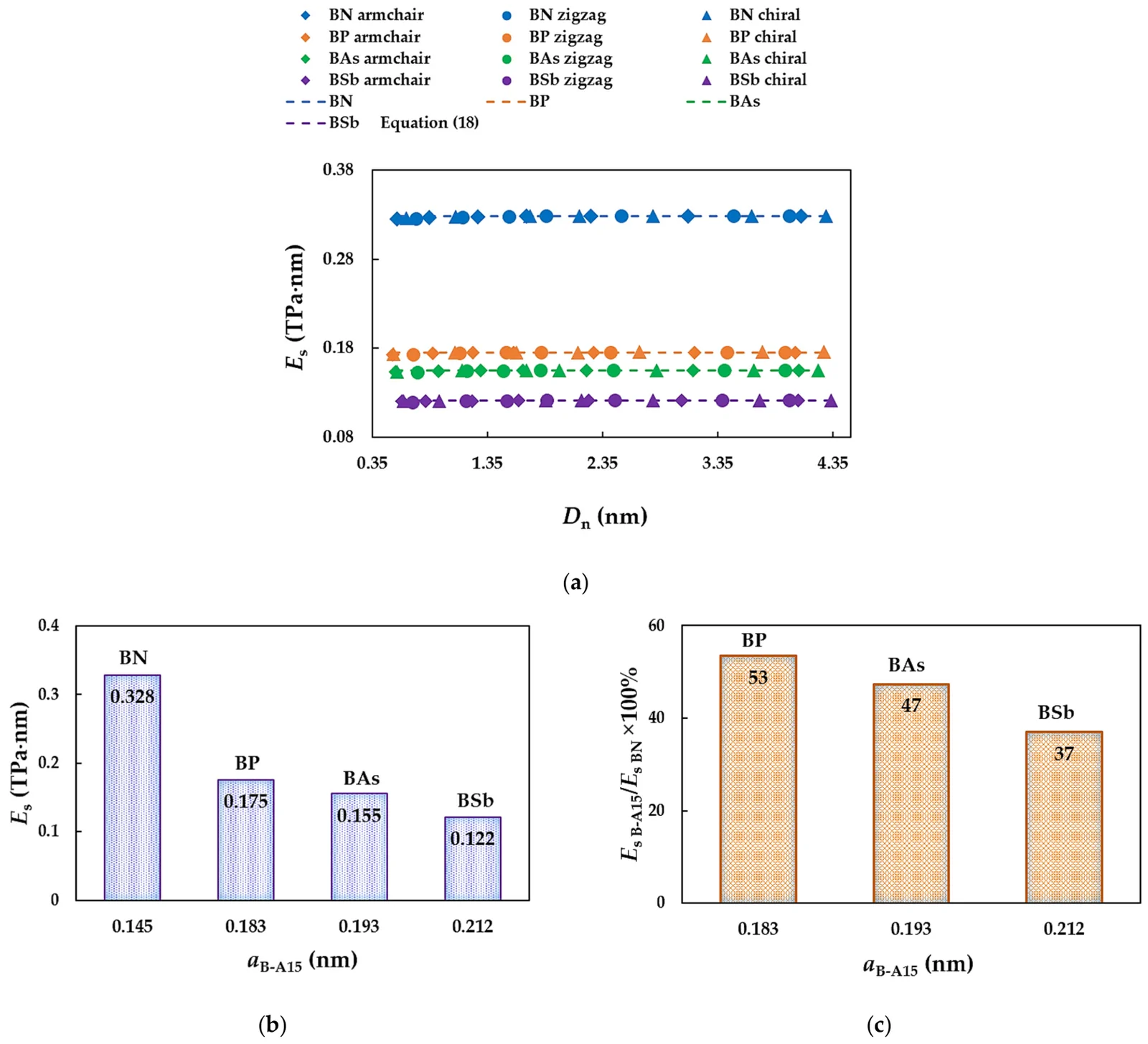
Surface Young’s modulus (*E*_s_) of SWBNNTs, SWBPNTs, SWBAsNTs and SWBSbNTs as a function of: (**a**) nanotube diameter (*D*_n_); (**b**) equilibrium bond length (*a*_B-A15_); and (**c**) normalized *E*_s_ values of SWBPNTs, SWBAsNTs and SWBSbNTs relative to SWBNNTs.

The surface Young’s modulus obtained from Equation (13) is plotted as a function of the nanotube diameter in Figure 4a, together with the analytical values calculated from Equation (18). Regardless of nanotube chirality and the boron–group 15 compound considered, the surface Young’s modulus remains practically constant over the investigated diameter range, demonstrating its independence from nanotube diameter. The excellent agreement between the analytical predictions and the numerical results is evident in Figure 4a. The mean relative difference between the analytical and numerical values of *E*_s_ is approximately 0.10% for BN, BP and BSb nanotubes, and approximately 0.20% for BAs nanotubes. Consequently, Equation (18) allows the surface Young’s modulus of boron–group 15 nanotubes to be accurately evaluated within the investigated diameter range.

To investigate the influence of the group 15 atoms (N, P, As and Sb) on the surface Young’s modulus, the analytical values are summarized in Figure 4b as a function of the equilibrium bond length. A gradual decrease in *E*_s_ is observed with increasing bond length, indicating a progressive reduction in the surface Young’s modulus from BNNTs to BSbNTs. To facilitate the comparison among the investigated nanotubes, Figure 4c presents the surface Young’s modulus relative to that of BNNTs, which were chosen as the reference because they have the highest surface Young’s modulus within the boron–group 15 nanotubes family and elastic properties comparable to those of carbon nanotubes. The most pronounced reduction occurs for BPNTs, whereas further substitution of the group 15 atoms leads to a more moderate decrease in *E*_s_. The corresponding values for BPNTs, BAsNTs and BSbNTs are approximately 53%, 47% and 37% of that obtained for BNNTs, respectively. These results may assist in the selection of suitable nanotube compounds for nanodevice applications that require a balance between mechanical performance and material properties. It should be noted that a comparable increase in the equilibrium bond length for group 13–nitride nanotubes [42] corresponds to a considerably higher reduction in the normalized surface Young’s modulus, with the value for *E*_s_ for thallium nitride nanotubes (TlNNTs) being approximately 17% of that of BNNTs. This observation further confirms that the elastic response is affected differently by substitution of the group 15 atoms in boron–group 15 nanotubes than by substitution of the group 13 atoms in nitride nanotubes.

**Figure 5:**
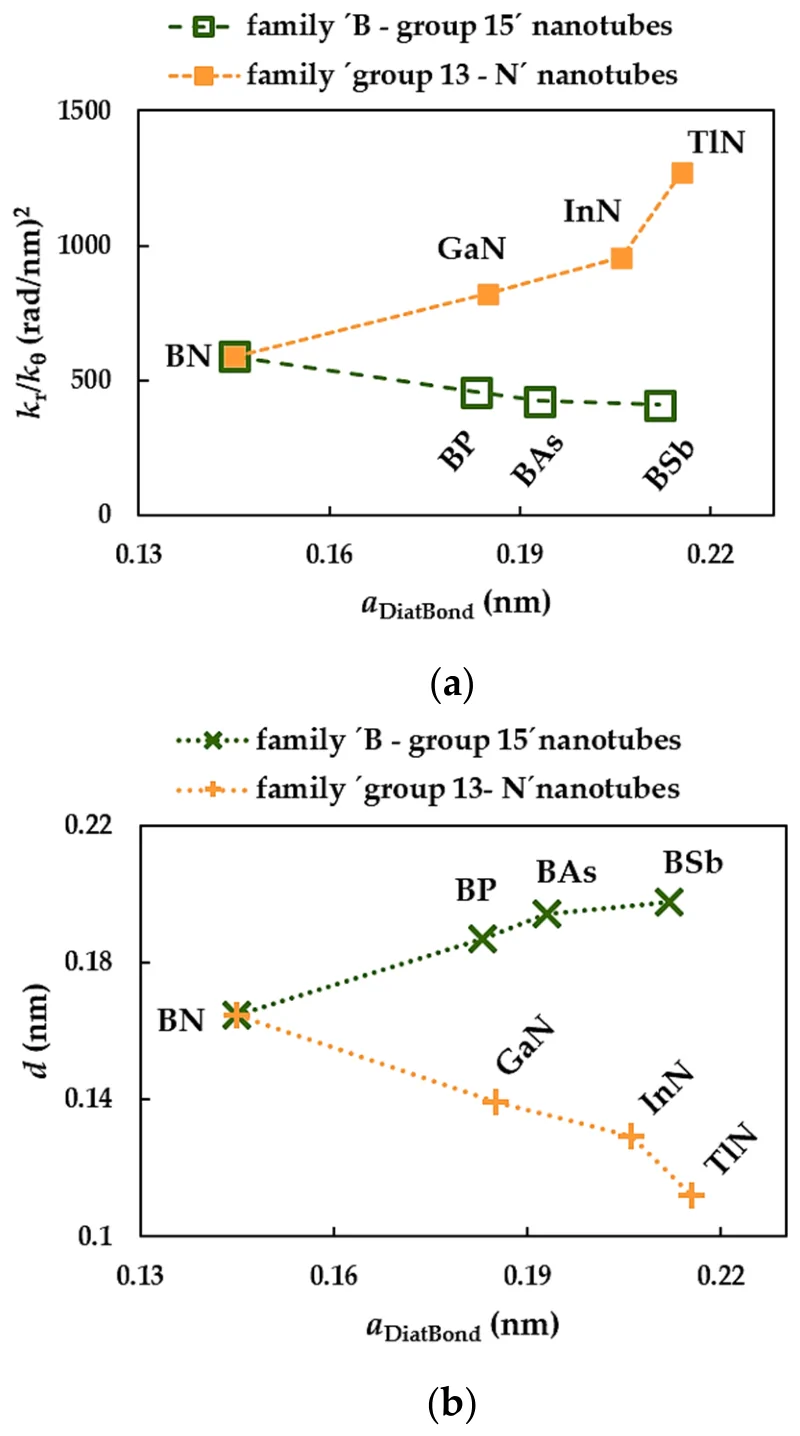
(**a**) Ratio between the bond-stretching and bond-bending force constants, *k*_r_/*k*_θ_, and (**b**) equivalent beam diameter, *d*, as functions of the equilibrium bond length for boron–group 15 and group 13–nitride nanotubes. The *k*_r_ and *k*_θ_ values for the group 13–nitride nanotubes were taken from Ref. [42].

The different elastic responses observed for the boron–group 15 and group 13–nitride nanotube families cannot be explained solely by differences in the equilibrium bond length, since both families cover a comparable bond-length range. An explanation may lie in the different evolution of the equivalent beam characteristics derived from the corresponding molecular bond characteristics, as represented by the force field constants. Within the modelling framework adopted in the present study, the diatomic bond is represented by the corresponding force field constants, from which the geometrical and elastic properties of the equivalent beam elements used in the finite element simulations are derived (Table 3). To further examine the influence of the molecular bond on the elastic response, the ratio between the bond-stretching and bond-bending force field constants (*k*_r_/*k*_θ_) is plotted in Figure 5a as a function of the equilibrium bond length for both nanotube families.

For boron–group 15 nanotubes, the ratio of (*k*_r_/*k*_θ_) decreases, albeit moderately, with increasing bond length, while a considerable increase is found for the group 13–nitride nanotubes. Since the equivalent beam diameter (*d*) is determined by this ratio, $d=4\sqrt{k_{\theta }/k_{r}}$, these opposite trends lead to contrasting variations in the equivalent beam geometry, as shown in Figure 5b. It should be noted that *d* is an equivalent geometric parameter derived from the molecular force constants and does not represent the physical diameter of the chemical bond; its evolution reflects, within the NCM/MSM formulation, the relative stretching and bending resistance assigned to the corresponding molecular bonds. The equivalent beam diameter progressively increases from BNNTs to BSbNTs, while it decreases from BNNTs to TlNNTs. This interpretation also suggests that the increase in equivalent beam diameter across the boron–group 15 nanotube family may partially mitigate the reduction in the surface Young’s modulus associated with increasing bond length while the opposite trend observed for the group 13–nitride nanotubes tends to reinforce this reduction. Consequently, the opposite evolution of the equivalent beam characteristics provides a plausible explanation for the substantially different rates at which the surface Young’s modulus decreases with increasing bond length in the two nanotube families.

Finally, the surface Young’s modulus values obtained in the present study were compared with data available in the literature, as summarized in Table 5. For SWBNNTs, the greater availability of published data allows for a more detailed comparison with both theoretical and experimental results. The latter include the studies of Zhou et al. [43] and Chen et al. [32], who evaluated the Young’s modulus of multiwalled BNNTs using, respectively, a high-order resonance technique in high-resolution transmission electron microscopy (HRTEM) and in situ axial compression in a transmission electron microscope (TEM).

The available data are considerably more limited for the other nanotubes investigated in the present study. Therefore, for completeness, the results for the corresponding two-dimensional nanostructures are also included for BP, BAs and BSb, providing additional reference values where nanotube data are scarce or unavailable. The results selected for comparison were obtained using molecular dynamics (MD) simulations with different potential functions to describe the interatomic interactions, including the Tersoff–Brenner (TB) [29,44] and second-generation reactive empirical bond-order (REBO) [45] potentials; also included are results obtained by: density functional theory (DFT) calculations using the local density approximation (LDA) [46]; continuum mechanics (CM) [31]; and NCM/MSM approaches [30,33].

When the literature studies report the conventional Young’s modulus *E* for BNNTs, the corresponding surface Young’s modulus values, *E*_s_ shown in Table 5, were evaluated from *E*_s_ = *Et*_n_, using the nanotube wall thickness adopted in the respective studies. For the experimental results of Zhou et al. [43] and Chen et al. [32], *t*_n_ = 0.34 nm was adopted, corresponding to the commonly used value for BNNTs [21].

**Table 5:** Comparison of the present surface Young’s modulus values with modelling and experimental results available in the literature.

Reference	Compound	Method	*E*_s_ (TPa·nm)		Difference, %	Comments
			Reference	Current		
Santosh et al. [47]	BN	MD: force constant approach	0.346 *	0.328	5.0	converged average value for (n, n) and (n, 0) NTs with *D*_n_ ranging from 1.385 to 4.038 nm
Tao et al. [29]		MD: TB potential + FE model	0.316 *		3.8	converged average value for (n, 0) NTs with *D*_n_ in a range of 0.86 to 1.38 nm
Kumar et al. [45]		MD: REBO potential	0.329 *		0.2	converged average value for (n, n) NTs with *D*_n_ ranging from 1.383 to 2.767 nm
Oh [31]		CM: continuum lattice thermodynamic + TB potential	0.322 *		2.0	converged average value for (n, 0) NTs with *D*_n_ ranging from 1.8 to 4.0 nm
Yan et al. [30]		NCM/MSM: longitudinal and torsional vibrations	0.324 *		1.4	converged average value for (n, n) and (n, 0) NTs with *D*_n_ ranging from 1.404 to 3.930 nm
Zhou et al. [43]		Experimental: HRTEM (in situ high order resonance)	0.308 *		6.6	Average *E*_s_ value for multiwalled BNNTs with outer NTs diameters ranging from 32.3 to 56.7 nm
Chen et al. [32]		Experimental: TEM (in situ compression)	0.357 *		8.0	multiwalled BNNTs with 40 layers and *D*_n_ = 10.30 nm
Jiang and Guo [33]		NCM/MSM: analytical solution	0.277		18.5	converged average value for (n, n) and (n, 0) NTs with *D*_n_ ranging from 0.512 to 1.881 nm
	BP		0.118	0.175	48.7	converged average value for (n, n) and (n, 0) NTs with *D*_n_ ranging from 0.429 to 2.759 nm
Singh et al. [44]		MD: TB potential	0.140		25.3	2D monolayer with dimensions of 10.3891 × 12.2105 nm^2^
Zhuang and Hennig [46]		DFT: LDA	0.172		2.0	2D monolayer
	BAs		0.146	0.155	6.5	
	BSb		0.119	0.122	2.1	
Le [48]		MD: harmonic force field parameters	0.095		27.9	

* *E*_s_ was calculated from the reported Young’s modulus *E* using *t*_n_ = 0.33 nm for Kumar et al. [45] and Oh [31], *t*_n_ = 0.333 nm for Yan et al. [30], and *t*_n_ = 0.34 nm for Santosh et al. [47] and Tao et al. [29], as adopted in the respective studies. The latter value, commonly adopted for BNNTs [21], was also used to calculate *E*_s_ from the experimental results of Zhou et al. [43] and Chen et al. [32].

As shown in Table 5, the surface Young’s modulus obtained for SWBNNTs is consistent with the range of theoretical and experimental results reported in the literature. Most of the selected values differ by less than 8% from the present *E*_s_ result, whereas the NCM/MSM result by Jiang and Guo [33] shows a larger deviation of 18.5%. The availability of outcomes obtained using different modelling approaches, together with available experimental measurements, provides a useful benchmark for the calculated SWBNNT surface Young’s modulus and highlights the dispersion among the results reported in the literature.

For BP, BAs and BSb, the considerably more limited availability of literature data restricts the extent of the comparison. Among the selected results, the closest values to those obtained in the present study are provided by the DFT calculations of Zhuang and Hennig [46] for the corresponding monolayers, with deviations of 2.0%, 6.5% and 2.1% for BP, BAs and BSb, respectively. These comparisons should nevertheless be interpreted with caution, because the reference results concern two-dimensional structures rather than nanotubes.

Larger discrepancies are observed when compared with other available results. The MD simulations of Singh et al. [44], who employed the TB potential to describe the interactions between boron and phosphorus atoms, yielded a surface Young’s modulus approximately 25% lower than that obtained in the present study. An even larger difference is found with the analytical NCM/MSM model proposed by Jiang and Guo [33], in which the B–P covalent bonds are represented by equivalent spring elements. It is worth noting that, for BNNTs, the results reported by Jiang and Guo [33] are considerably closer to the present values. Although both studies employ the NCM/MSM framework, this comparison illustrates that the modelling approach alone does not necessarily lead to similar predictions, as the results are also influenced by the adopted assumptions and modelling parameters. Similarly, although Le [48] adopted force field constants close to those used in the present study, the corresponding MD simulations yielded a considerably lower surface Young’s modulus for the BSb monolayer than that obtained here for BSbNTs.

Overall, Table 5 shows a considerable scatter among the literature values and varying degrees of consistency with the present results. For BNNTs the comparison is supported by both theoretical and experimental data; however, for single-walled BPNTs, BAsNTs, and BSbNTs, the available theoretical data remain too limited and dispersed to establish reliable reference values for the surface Young’s modulus. This highlights the need for further investigations. Moreover, differences in dimensionality, modelling approaches, and parameterizations across the available studies mean that these comparisons should be regarded as indicative rather than as a direct quantitative validation of the present results.

### 3.3. Surface Shear Modulus and Poisson’s Ratio of Boron–Group 15 Nanotubes

In this section, the surface shear modulus (*G*_s_) and Poisson’s ratio (*ν*) of boron–group 15 nanotubes are investigated. These properties complement the elastic characterization presented in the previous sections and complete the description of the nanotubes’ elastic behaviour.

The surface shear modulus of SWBNNTs, SWBPNTs, SWBAsNTs and SWBSbNTs was evaluated using Equation (14), which combines the tensile, bending and torsional rigidities obtained from the finite element analyses. Since these rigidities are accurately described by the analytical expressions derived in Section 3.1, a diameter-independent expression for the surface shear modulus can be readily obtained by combining Equations (14)–(17):

(19) $$G_{s}=\frac{\gamma_{\mathrm{B-A}15}}{\pi \sqrt{32{\left(\frac{\beta_{\mathrm{B-A}15}}{\alpha_{\mathrm{B-A}15}}\right)}^{3}}}.$$

where *α*_B-A15_, *β*_B-A15_ and *γ*_B-A15_ are the fitting parameters from Table 4 corresponding to the tensile, bending and torsional rigidities, respectively.

**Figure 6:**
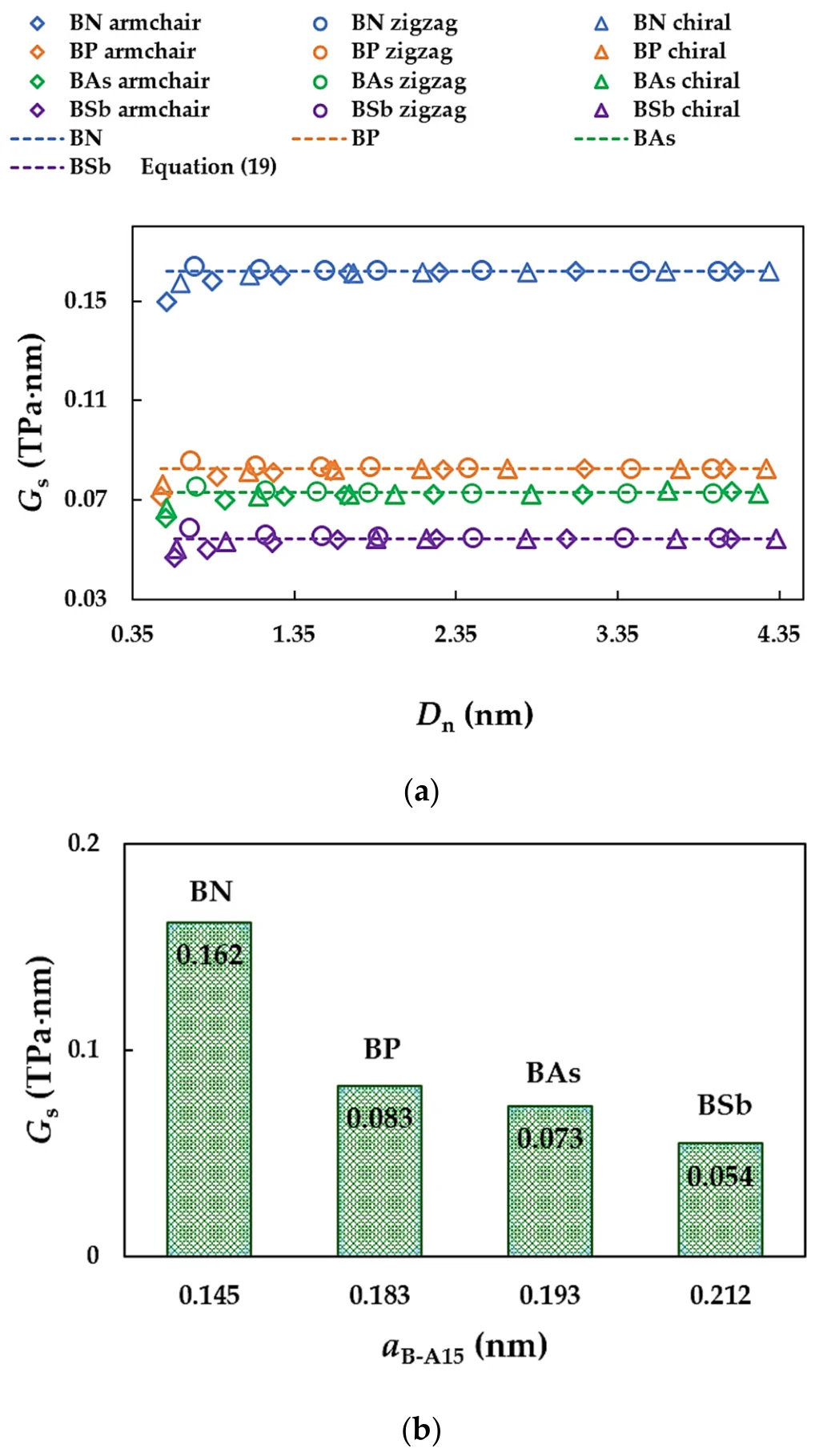
Surface shear modulus (*G*_s_) of SWBNNTs, SWBPNTs, SWBAsNTs and SWBSbNTs as a function of: (**a**) nanotube diameter (*D*_n_); (**b**) equilibrium bond length (*a*_B-A15_).

The surface shear modulus (*G*_s_) obtained from Equation (14) is plotted as a function of the nanotube diameter (*D*_n_) in Figure 6a (symbols), together with the analytical values calculated from Equation (19) (dashed lines). Unlike the surface Young’s modulus, which remains practically independent of nanotube diameter, the surface shear modulus exhibits a weak diameter dependence for nanotubes with *D*_n_ ≲ 1.1 nm. Within this range, a slight decrease in *G*_s_ is observed for zigzag nanotubes, whereas armchair and chiral nanotubes exhibit a modest increase. With increasing nanotube diameter, the surface shear modulus gradually converges towards an approximately constant value, irrespective of nanotube chirality and the boron–group 15 compound considered.

This behaviour differs from that previously reported for nitride-group 13 nanotubes [40], where the threshold diameter beyond which *G*_s_ becomes nearly constant exhibits a clear increasing trend with the equilibrium bond length. For the boron–group 15 nanotubes investigated in the present work, no such relationship can be identified. Instead, the results suggest that nanotubes with *D*_n_ ≳ 1.1 nm may be considered nearly diameter-independent with respect to the surface shear modulus. Consequently, Equation (19) provides an accurate analytical prediction of *G_S_* over this diameter range. The mean relative difference between the analytical and numerical values remains below 0.10% for all boron–group 15 nanotubes, except for BAsNTs, for which it is still below 0.50%.

The influence of the group 15 atoms on the surface shear modulus is illustrated in Figure 6b, where the analytical values of *G*_s_ are plotted as a function of the equilibrium bond length. A pronounced reduction in the surface shear modulus is observed when moving from BNNTs to BPNTs, with *G*_s_ decreasing by approximately one half. As the bond length increases further, the decrease becomes progressively less pronounced, leading to a more gradual reduction in the surface shear modulus from BPNTs to BSbNTs.

This behaviour contrasts with that reported for the group 13–nitride nanotube family [42], where a comparable increase in the equilibrium bond length results in a substantially greater reduction in the surface shear modulus. Indeed, the *G*_s_ value of TlNNTs is approximately 8.5 times lower than that of BNNTs, whereas the corresponding value for BSbNTs is only about three times lower than that of BNNTs. This observation further indicates that varying the group 15 atoms while keeping boron fixed affects the elastic response differently from varying the group 13 atom while keeping nitrogen fixed. A plausible explanation for this distinct behaviour lies in the different evolution of the equivalent beam characteristics derived from the corresponding molecular bond characteristics, as represented by the force field constants (see Section 3.3).

BNNTs are by far the most extensively investigated member of the boron–group 15 nanotube family. However, studies dedicated to their surface shear modulus remain remarkably limited, and the available theoretical results exhibit considerable scatter, as comprehensively discussed in the authors’ previous studies [21,42]. For BP, BAs and BSb nanostructures, whether nanotubes or monolayers, the available data are even scarcer, and in several cases, remain unavailable. The surface shear modulus reported by Singh et al. [44] for a boron phosphide monolayer is approximately 35% lower than the present value. However, *G*_s_ of nanotubes is derived from their torsional response, which has no direct counterpart in monolayers, and our previous study [49] showed that nanotubes exhibit higher surface shear moduli than the corresponding monolayers. Therefore, although the present analytical values provide useful reference data, reliable benchmark values require further investigations specifically addressing the torsional behaviour of boron–group 15 nanotubes.

The remaining elastic property involving torsional rigidity in its evaluation, namely the Poisson’s ratio (*ν*) of boron–group 15 nanotubes, is discussed in the following. The values were evaluated using Equation (12), which combines the bending and torsional rigidities, *EI* and *GJ*, obtained from the corresponding numerical simulations. Since both rigidities are accurately represented by the analytical expressions derived in the previous section, Equation (12) can be rewritten in analytical form by substituting Equations (16) and (17), together with the fitting parameters *β*_B-A15_ and *γ*_B-A15_ listed in Table 4, leading to the following expression independent of the nanotube diameter:

(20) $$\nu =\frac{\beta_{\mathrm{B-A}15}}{\gamma_{\mathrm{B-A}15}}-1.$$

**Figure 7:**
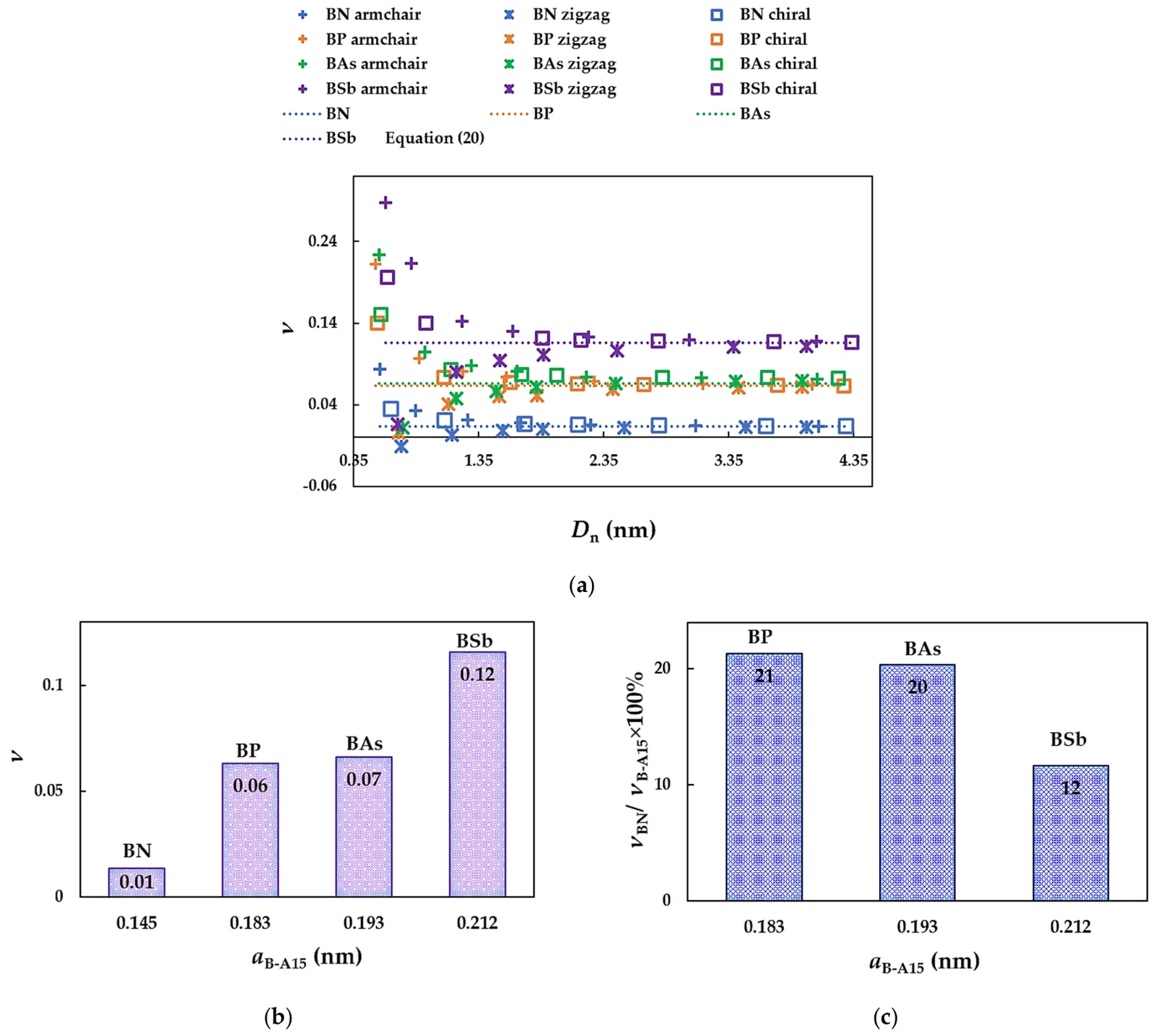
Poisson’s ratio (*ν*) of SWBNNTs, SWBPNTs, SWBAsNTs and SWBSbNTs as a function of: (**a**) nanotube diameter (*D*_n_); (**b**) equilibrium bond length (*a*_B-A15_); and (**c**) normalized Poisson’s ratio of SWBPNTs, SWBAsNTs and SWBSbNTs with respect to SWBNNTs.

The Poisson’s ratio (*ν*) obtained from Equation (12) is plotted as a function of the nanotube diameter (*D*_n_) in Figure 7a (symbols), together with the analytical values calculated from Equation (20) (dotted lines). For nanotube diameters up to approximately 2.1 nm, Poisson’s ratio exhibits different trends depending on the nanotube chirality, decreasing for armchair and chiral nanotubes, while a slight increase is observed for zigzag nanotubes. In all cases, these variations become more pronounced with increasing equilibrium bond length. At larger diameters, Poisson’s ratio gradually approaches an approximately constant value, regardless of the nanotube compound and chirality. Consequently, Equation (20) accurately predicts the Poisson’s ratio within this diameter-independent range, yielding mean relative differences between the analytical and numerical values not exceeding 0.4%. A similar convergence towards a diameter-independent Poisson’s ratio was previously observed for the group 13–nitride nanotube family [42]. However, for the group 13–nitride nanotube family, the onset of the diameter-independent regime shifts towards larger nanotube diameters with increasing equilibrium bond length [42].

Figure 7b summarizes the influence of the group 15 atoms on Poisson’s ratio by presenting the analytical values as a function of the equilibrium bond length. Poisson’s ratio increases progressively from the lowest value of 0.01 for BNNTs to 0.12 for BSbNTs. This evolution directly reflects the increasing *β*_B-A15_/*γ*_B-A15_ ratio shown in Figure 3c, from which Poisson’s ratio is analytically derived. Indeed, the bending (*EI*) and torsional (*GJ*) rigidities are nearly identical for BNNTs and remain relatively close throughout the boron–group 15 nanotube family, with the largest difference occurring for BSbNTs.

A different behaviour is observed for the group 13–nitride nanotube family, where Poisson’s ratio increases much more rapidly from approximately 0.01 for BNNTs to 0.50 for TlNNTs [42]. This trend is likewise reflected by the corresponding *β*/*γ* ratio, indicating a considerably larger divergence between the bending and torsional rigidities. Therefore, the different evolution of the Poisson’s ratio in the two nanotube families is consistent with the interpretation proposed for the elastic response mentioned above, further supporting the role of the equivalent beam characteristics derived from the corresponding molecular bond properties, as represented by the force field constants.

To facilitate the comparison among the investigated nanotubes, Poisson’s ratio of SWBPNTs, SWBAsNTs and SWBSbNTs was also normalized with respect to that of SWBNNTs, as shown in Figure 7c. Poisson’s ratio of BNNTs corresponds to approximately 21%, 20% and 12% of the values obtained for BPNTs, BAsNTs and BSbNTs, respectively. Unlike the monotonic reduction observed for the surface Young’s and shear moduli, the normalized Poisson’s ratio exhibits a distinct behaviour, with similar values for BPNTs and BAsNTs followed by a more pronounced increase for BSbNTs. These differences may become particularly relevant in the design of nanotube-based heterostructures, where the compatibility of elastic deformation between adjacent constituents is an important consideration.

Boron nitride NTs are by far the most extensively investigated member of the boron–group 15 nanotube family. Nevertheless, the available Poisson’s ratio data remain limited and scattered even for BNNTs, while corresponding results for BPNTs, BAsNTs and BSbNTs are particularly scarce or non-existent. The currently available data are therefore insufficient to ascertain reliable benchmark values, highlighting the need for further investigations of Poisson’s ratio of boron–group 15 nanotubes.

### 3.4. Outlook

The analytical expressions proposed in Equations (15)–(20) provide a compact description of the elastic behaviour of boron–group 15 nanotubes over the investigated diameter range, covering the tensile, bending and torsional rigidities as well as the surface Young’s and shear moduli, and Poisson’s ratio. In addition to offering an efficient alternative to finite element analyses, these relationships may also be incorporated into higher-level computational frameworks, enabling the elastic properties of nanotubes to be readily evaluated.

This capability may be particularly useful when nanotubes are considered as constituents of larger nanostructures, where their mechanical properties must be evaluated repeatedly. In such cases, the proposed relationships can be incorporated into multiscale models and optimization procedures, in which the elastic properties of the individual nanotubes are required. The analytical expressions can also facilitate the generation of large datasets covering different nanotube diameters and compositions without requiring a separate numerical simulation for each configuration. Such datasets could subsequently be used to develop physics-based surrogate or data-driven models for the mechanical response of more complex nanotube-based structures, thereby extending the range of systems that can be explored while reducing the computational cost associated with repeated numerical simulations.

A remaining challenge is the limited availability of force field constants for the less investigated boron–group 15 compounds, highlighting the importance of further efforts towards their reliable determination.

## 4. Conclusions

This study provides a systematic comparative assessment of the elastic behaviour of single-walled boron–group 15 nanotubes, including SWBNNTs, SWBPNTs, SWBAsNTs and SWBSbNTs, using the NCM/MSM approach. Particular attention is given to SWBAsNTs and SWBSbNTs, whose elastic behaviour remains largely unexplored, with available literature data being restricted mainly to the corresponding two-dimensional nanostructures rather than nanotubes. The consistent application of the same modelling framework to the four compounds allows for a direct comparison of their elastic response and enables the systematic evaluation of the effect of changing the atom in group 15, while keeping the boron fixed.

The results reveal distinct trends across the boron–group 15 nanotube family. The tensile, bending and torsional rigidities are accurately described over the investigated diameter range by the proposed analytical expressions, which also enable evaluation of the surface Young’s modulus, surface shear modulus and Poisson’s ratio. The surface Young’s modulus remains practically independent of nanotube diameter, whereas the surface shear modulus and Poisson’s ratio exhibit diameter-dependent behaviour before approaching nearly constant values. Along the BN-to-BSb sequence, the increase in the equilibrium B–A15 bond length is associated with a progressive reduction in both surface Young’s and shear moduli, with the most pronounced change occurring between BNNTs and BPNTs, while Poisson’s ratio increases across the family.

Comparison with the previously investigated group 13–nitride nanotube family shows that comparable variations in equilibrium bond length lead to markedly different changes in the elastic response. Within the equivalent beam model, a plausible explanation for this behaviour was found in the different evolution of the equivalent beam characteristics resulting from the corresponding molecular bond properties, as represented by the force field constants. In particular, the equivalent beam elements become progressively thicker across the boron–group 15 family and thinner across the group 13–nitride family, which may contribute to the different rates of reduction in the elastic moduli and increase in Poisson’s ratio observed with increasing bond length in the two nanotube families.

Overall, the present results broaden the available knowledge of the elastic behaviour of boron–group 15 nanotubes and, importantly, provide a consistent comparative description across the entire family, including the scarcely investigated BAsNTs and BSbNTs, for which the available literature data remain insufficient to define representative elastic properties. Thus, the main contribution of the present study lies in establishing, within a common modelling framework, the evolution and comparative trends of the elastic response across the entire family of group 15 boron nanotubes, which cannot be established from isolated studies of individual compounds. The analytical relationships developed in this work may also support potential multiscale and data-driven investigations of boron–group 15 nanotubes, enabling efficient incorporation of their elastic properties into more complex computational frameworks.

## Figures and Tables

**Table 1:** Geometrical characteristics of the boron–group 15 nanotubes considered in the present study.

NT	SWBNNTs		SWBPNTs		SWBAsNTs		SWBSbNTs	
	(*n*, *m*)	*D*_n_, nm *	(*n*, *m*)	*D*_n_, nm	(*n*, *m*)	*D*_n_, nm	(*n*, *m*)	*D*_n_, nm
zigzag, *θ* = 0°	(9, 0)	0.729	(7, 0)	0.706	(7, 0)	0.745	(6, 0)	0.701
	(14, 0)	1.135	(11, 0)	1.110	(11, 0)	1.170	(10, 0)	1.169
	(19, 0)	1.540	(15, 0)	1.513	(14, 0)	1.490	(13, 0)	1.519
	(23, 0)	1.864	(18, 0)	1.816	(17, 0)	1.809	(16, 0)	1.870
	(31, 0)	2.512	(24, 0)	2.421	(23, 0)	2.447	(21, 0)	2.455
	(43, 0)	3.485	(34, 0)	3.430	(32, 0)	3.405	(29, 0)	3.390
	(49, 0)	3.971	(39, 0)	3.935	(37, 0)	3.937	(34, 0)	3.974
chiral, *θ* = 19.1°	(6, 3)	0.643	(4, 2)	0.534	(4, 2)	0.563	(4, 2)	0.618
	(10, 5)	1.072	(8, 4)	1.068	(8, 4)	1.126	(6, 3)	0.928
	(16, 8)	1.715	(12, 6)	1.602	(12, 6)	1.689	(12, 6)	1.855
	(20, 10)	2.144	(16, 8)	2.136	(14, 7)	1.971	(14, 7)	2.165
	(26, 13)	2.788	(20, 10)	2.669	(20, 10)	2.815	(18, 9)	2.783
	(34, 17)	3.645	(28, 14)	3.737	(26, 13)	3.660	(24, 12)	3.711
	(40, 20)	4.289	(32, 16)	4.271	(30, 15)	4.223	(28, 14)	4.329
armchair, *θ* = 30°	(4, 4)	0.561	(3, 3)	0.524	(3, 3)	0.553	(3, 3)	0.607
	(6, 6)	0.842	(5, 5)	0.874	(5, 5)	0.922	(4, 4)	0.810
	(9, 9)	1.263	(7, 7)	1.223	(7, 7)	1.290	(6, 6)	1.215
	(12, 12)	1.684	(9, 9)	1.573	(9, 9)	1.659	(8, 8)	1.620
	(16, 16)	2.246	(13, 13)	2.272	(12, 12)	2.212	(11, 11)	2.227
	(22, 22)	3.088	(18, 18)	3.146	(17, 17)	3.133	(15, 15)	3.037
	(29, 29)	4.071	(23, 23)	4.019	(22, 22)	4.055	(20, 20)	4.049

* The diameters, *D*_n_, of SWBNNTs, are calculated adopting the bond length *a*_B-N_ = 0.147 nm, as defined by Nanotube Modeler© software; for the SWBPNTs, SWBAsNTs and SWBSbNTs, the bond lengths *a*_B-P_ = 0.183 nm [35], *a*_B-As_ = 0.193 nm [35] and *a*_B-Sb_ = 0.212 nm [35] were respectively assumed.

**Table 2:** Material properties and force field constants adopted for the boron–group 15 nanotubes.

Compound	*a*_P-A15_, nm [35]	*E*_s_*,* nN/nm [35]	*ν* [35]	*k*_r_, nN/nm	*k*_θ_, nN·nm/rad^2^	*k*_τ_, nN·nm/rad^2^
BN	0.145	267	0.21	585	0.994	2.470 ^1^
BP	0.183	135	0.28	325	0.709	0.625 ^2^
BAs	0.193	119	0.29	290	0.684	
BSb	0.212	91	0.34	239	0.584	

^1^ Values from Ansari et al. [39]; ^2^ Values acquired using the DREIDING force field [38].

**Table 3:** Geometrical and elastic properties of the equivalent beam elements used in the finite element simulations.

Compound	Diameter, *d*, nm	Formulation	Young’s Modulus, *E*_b_, GPa	Formulation	Shear Modulus, *G*_b_, GPa	Formulation	Poisson’s Ratio, *ν*_b_ [35]
BN	0.1648	$d=4\sqrt{\frac{k_{\theta }}{k_{r}}}$	3977	$E_{b}=\frac{k_{r}^{2}l}{4\pi k_{\theta }}$	4941	$G_{b}=\frac{{k_{r}^{2}k}_{\tau }l}{8\pi k_{\theta }^{2}}$	0.21
BP	0.1869		2165		954		0.28
BAs	0.1942		1892		864		0.29
BSb	0.1979		1646		880		0.34

**Table 4:** Fitting parameters *α*_B-A15_, *β*_B-A15_ and *γ*_B-A15_ and mean relative difference between the rigidities obtained analytically from Equations (15)–(17) and the corresponding finite element results.

Compound	Fitting Parameters			Mean Difference ^2^, %		
	*α*_B-A15_, nN/nm	*β*_B-A15_, nN/nm	*γ*_B-A15_, nN/nm	*EA*, nN	*EI*, nN·nm^2^	*GJ*, nN·nm^2^
BN	1031.04 ^1^	128.77 ^1^	127.06 ^1^	0.18	0.64	0.32
BP	551.04 ^1^	68.84 ^1^	64.75 ^1^	0.35	0.67	0.53
BAs	486.87	60.45	56.7	0.45	0.88	0.65
BSb	381.7	47.69	42.74	0.49	0.85	0.65

^1^ Values of the fitting parameters for the BNNTs and BPNTs are similar to those obtained in the author’s previous works [21,34]; ^2^ The mean relative difference was calculated as the average of the values of the relative differences between the analytical predictions and the corresponding finite element rigidities.

## Data Availability

The original contributions presented in this study are included in the article/Supplementary Material. Further inquiries can be directed to the corresponding author.

## Supplementary Materials

The following supporting information can be downloaded at: https://www.mdpi.com/article/10.3390/ma19214483/s1, Figure S1: Boundary and loading conditions adopted in the tensile, bending and torsion tests; Table S1: Numerical values of the tensile, bending and torsional rigidities for the investigated nanotube configurations.

## Abbreviations

The following abbreviations are used in this manuscript:

BNNT	Boron nitride nanotube
BPNT	Boron phosphide nanotube
BAsNT	Boron arsenide nanotube
BSbNT	Boron antimonide nanotube
NCM	Nanoscale Continuum Modelling
MSM	Molecular Structural Mechanics
2D	Two-dimensional
BN	Boron nitride
BP	Boron phosphide
BAs	Boron arsenide
BSb	Boron antimonide
1D	One-dimensional
NT	Nanotube
SWBNNT	Single-walled boron nitride nanotube
SWBPNT	Single-walled boron phosphide nanotube
SWBASNT	Single-walled boron arsenide nanotube
SWBSbNT	Single-walled boron antimonide nanotube
FE	Finite element
TlN	Thallium nitride
TlNNT	Thallium nitride nanotube
HRTEM	High Resolution Transmission Electron Microscopy
TEM	Transmission Electron Microscopy
MD	Molecular Dynamics
TB	Tersoff–Brenner
REBO	Reactive Empirical Bond Order
DFT	Density Functional Theory
LDA	Local Density Approximation
CM	Continuum Mechanics
