# Supplementary material for: Numerical Simulation Study on the Elastic Behaviour of Single-Walled Boron–Group 15 Nanotubes: A Comparative Assessment
Source: Materials (Basel). 2026 Oct 24;19(21):4483. doi: 10.3390/ma19214483 (PMC13764031; doi:10.3390/ma19214483)
Supplement: Supplementary file 1 [file materials-19-04483-s001.zip › materials-4576309-supplementary.pdf]

# Supplementary Materials

## Finite element implementation

The finite element (FE) analyses were performed using ABAQUS 2020/Standard. The diatomic bonds were represented by two-node, three-dimensional Euler–Bernoulli beam elements (B33), with circular cross-sections and linear elastic material properties. A single Static, General analysis step was used, with NLGEOM disabled and the default increment and convergence tolerance settings. At the fixed end, all six degrees of freedom of the edge nodes were constrained. For the tensile and bending tests, the total axial and transverse forces, respectively, were uniformly distributed among the nodes at the loaded end. For the torsion test, the nodes at the loaded end were kinematically coupled to a reference point, at which the torsional moment was applied, ensuring uniform transfer of the rotation. Under these linear elastic conditions, all analyses converged in a single increment. The numbers of nodes and elements vary with the nanotube geometry, since the FE discretization directly follows the atomic structure, with nodes representing atoms and beam elements representing the corresponding interatomic bonds.

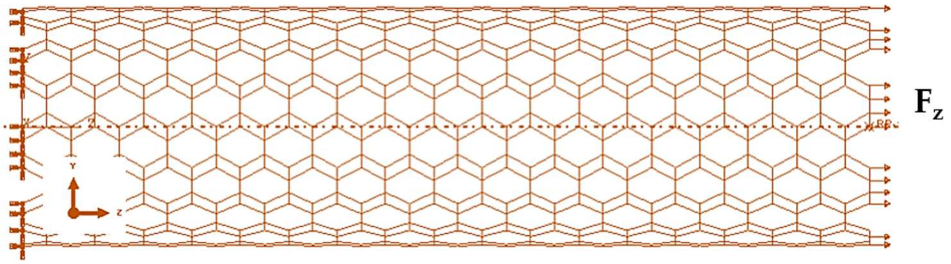


(**a**)

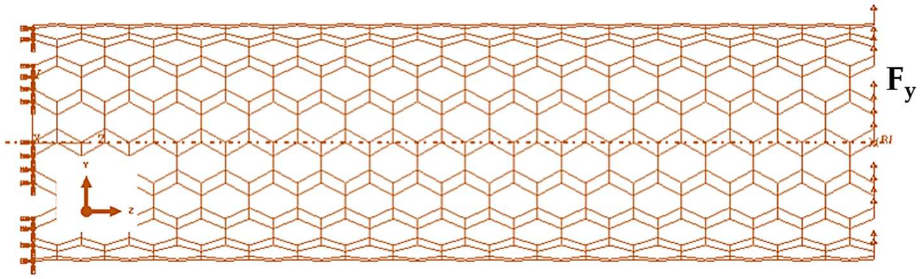


(**b**)

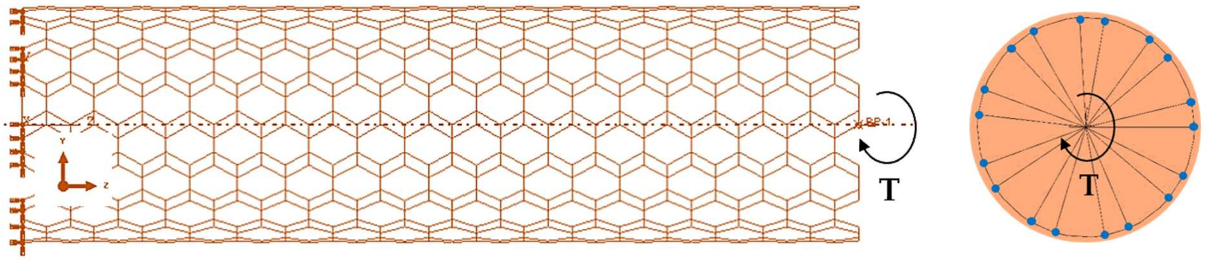


(**c**)

**Figure S1.** Boundary and loading conditions adopted in the (**a**) tensile, (**b**) bending and (**c**) torsion tests.

**Table S1.** Numerical values of the tensile, bending and torsional rigidities for the investigated nanotube configurations.

| NTs | n | m | $D_n$, nm | EA, nN | EI, nN·nm² | GJ, nN·nm² |
|---|---|---|---|---|---|---|
| SWBNNTs | 4 | 4 | 0.561 | 579.61728 | 23.38213 | 21.58380 |
| | 6 | 3 | 0.643 | 664.37393 | 35.01874 | 33.82435 |
| | 9 | 0 | 0.729 | 741.77234 | 49.08531 | 49.63942 |
| | 6 | 6 | 0.842 | 869.35343 | 78.00442 | 75.55021 |
| | 10 | 5 | 1.072 | 1107.61927 | 160.30786 | 157.02395 |
| | 14 | 0 | 1.135 | 1163.16186 | 186.59638 | 186.06070 |
| | 9 | 9 | 1.263 | 1303.22182 | 261.12401 | 255.79832 |
| | 19 | 0 | 1.540 | 1582.62813 | 467.98227 | 464.38872 |
| | 12 | 12 | 1.684 | 1737.23513 | 617.18276 | 606.62150 |
| | 16 | 8 | 1.715 | 1771.24058 | 653.19271 | 642.50965 |
| | 23 | 0 | 1.864 | 1918.26530 | 831.12175 | 822.99837 |
| | 20 | 10 | 2.144 | 2213.51482 | 1273.92548 | 1254.30347 |
| | 16 | 16 | 2.246 | 2316.21046 | 1460.79201 | 1438.97110 |
| | 31 | 0 | 2.512 | 2587.48077 | 2037.40424 | 2014.65018 |
| | 26 | 13 | 2.788 | 2876.88783 | 2795.34859 | 2754.56843 |
| | 22 | 22 | 3.088 | 3184.37157 | 3793.67015 | 3741.04358 |
| | 43 | 0 | 3.485 | 3591.25860 | 5441.09556 | 5375.14391 |
| | 34 | 17 | 3.645 | 3761.26634 | 6245.74921 | 6158.82069 |
| | 49 | 0 | 3.971 | 4092.80375 | 8052.60118 | 7955.00020 |
| | 29 | 29 | 4.071 | 4197.35903 | 8684.62650 | 8568.92651 |
| | 40 | 20 | 4.289 | 4424.56717 | 10166.09238 | 10024.69307 |
| SWBPNTs | 3 | 3 | 0.524 | 289.86448 | 10.30162 | 8.49800 |
| | 4 | 2 | 0.534 | 293.47635 | 10.64813 | 9.33892 |
| | 7 | 0 | 0.706 | 378.78842 | 23.15771 | 23.03087 |
| | 5 | 5 | 0.874 | 482.50455 | 46.71202 | 42.58705 |
| | 8 | 4 | 1.068 | 589.64576 | 84.67708 | 78.83305 |
| | 11 | 0 | 1.110 | 604.92983 | 92.34164 | 88.76428 |
| | 7 | 7 | 1.223 | 675.19868 | 127.25573 | 117.71731 |
| | 15 | 0 | 1.513 | 829.10166 | 236.07941 | 224.74596 |
| | 9 | 9 | 1.573 | 867.85038 | 269.50959 | 250.91878 |
| | 12 | 6 | 1.602 | 884.24148 | 284.51906 | 266.40217 |
| | 18 | 0 | 1.816 | 996.62932 | 409.07813 | 389.33140 |
| | 16 | 8 | 2.136 | 1178.68775 | 673.05800 | 631.51706 |
| | 13 | 13 | 2.272 | 1253.18220 | 809.87307 | 757.93931 |
| | 24 | 0 | 2.421 | 1331.69920 | 972.77025 | 918.99775 |
| | 20 | 10 | 2.669 | 1473.07857 | 1313.09882 | 1233.08624 |
| | 18 | 18 | 3.146 | 1734.13064 | 2143.77279 | 2012.74632 |
| | 34 | 0 | 3.430 | 1888.52352 | 2770.86549 | 2612.63594 |
| | 28 | 14 | 3.737 | 2061.76322 | 3598.86320 | 3382.50759 |
| | 39 | 0 | 3.935 | 2167.08854 | 4183.91266 | 3942.71106 |
| | 23 | 23 | 4.019 | 2215.51631 | 4469.55206 | 4200.73369 |
| | 32 | 16 | 4.271 | 2356.07091 | 5370.24332 | 5048.81549 |

| NTs | n | m | $D_n$, nm | EA, nN | EI, nN·nm[2] | GJ, nN·nm[2] |
|---|---|---|---|---|---|---|
| SWBAsNTs | 3 | 3 | 0.553 | 270.50672 | 10.67145 | 8.71937 |
| | 4 | 2 | 0.563 | 273.96501 | 11.03547 | 9.59168 |
| | 7 | 0 | 0.745 | 353.45110 | 23.98938 | 23.71399 |
| | 5 | 5 | 0.922 | 450.57415 | 48.49274 | 43.90952 |
| | 8 | 4 | 1.126 | 550.53494 | 87.89357 | 81.14346 |
| | 11 | 0 | 1.170 | 564.69665 | 95.79230 | 91.41065 |
| | 7 | 7 | 1.290 | 630.16978 | 131.94692 | 121.30465 |
| | 14 | 0 | 1.490 | 721.79724 | 198.90405 | 188.23357 |
| | 9 | 9 | 1.659 | 809.89207 | 279.42612 | 258.42398 |
| | 12 | 6 | 1.689 | 825.63143 | 295.41673 | 274.20245 |
| | 17 | 0 | 1.809 | 878.43874 | 357.45770 | 336.85093 |
| | 14 | 7 | 1.971 | 963.14660 | 468.58551 | 435.40353 |
| | 12 | 12 | 2.212 | 1079.58465 | 660.77754 | 613.79966 |
| | 23 | 0 | 2.447 | 1191.41480 | 888.61405 | 833.35964 |
| | 20 | 10 | 2.815 | 1375.45430 | 1363.60502 | 1269.51555 |
| | 17 | 17 | 3.133 | 1529.16947 | 1875.67400 | 1746.83185 |
| | 32 | 0 | 3.405 | 1659.41573 | 2398.36263 | 2244.57770 |
| | 26 | 13 | 3.660 | 1771.40750 | 2911.98029 | 2763.38162 |
| | 37 | 0 | 3.937 | 1919.34916 | 3709.35025 | 3469.34384 |
| | 22 | 22 | 4.055 | 1964.85372 | 3977.43053 | 3760.78915 |
| | 30 | 15 | 4.223 | 2062.54944 | 4596.04477 | 4283.54110 |
| SWBSbNTs | 3 | 3 | 0.607 | 232.70878 | 11.00545 | 8.54417 |
| | 4 | 2 | 0.618 | 234.76015 | 11.26271 | 9.41454 |
| | 6 | 0 | 0.701 | 256.69764 | 15.13626 | 14.89689 |
| | 4 | 4 | 0.810 | 309.88841 | 25.74522 | 21.22095 |
| | 6 | 3 | 0.928 | 354.49858 | 38.46698 | 33.73815 |
| | 10 | 0 | 1.169 | 439.47593 | 73.88447 | 68.43378 |
| | 6 | 6 | 1.215 | 463.39445 | 86.35030 | 75.60401 |
| | 13 | 0 | 1.519 | 574.93525 | 164.25310 | 150.17252 |
| | 8 | 8 | 1.620 | 619.23556 | 203.77035 | 180.27092 |
| | 12 | 6 | 1.855 | 709.77694 | 306.10241 | 272.90306 |
| | 16 | 0 | 1.870 | 709.84273 | 308.07687 | 279.91116 |
| | 14 | 7 | 2.165 | 828.02768 | 485.69932 | 433.74597 |
| | 11 | 11 | 2.227 | 851.12932 | 528.18586 | 470.37544 |
| | 21 | 0 | 2.455 | 934.11553 | 699.87367 | 632.52159 |
| | 18 | 9 | 2.783 | 1064.40629 | 1031.15970 | 921.88634 |
| | 15 | 15 | 3.037 | 1160.26810 | 1337.06956 | 1194.58816 |
| | 29 | 0 | 3.390 | 1292.35572 | 1849.22471 | 1664.69332 |
| | 24 | 12 | 3.711 | 1418.88523 | 2442.00516 | 2185.20709 |
| | 34 | 0 | 3.974 | 1515.84822 | 2983.07079 | 2682.84877 |
| | 20 | 20 | 4.049 | 1546.86566 | 3166.94711 | 2833.79133 |
| | 28 | 14 | 4.329 | 1655.18224 | 3876.30950 | 3470.49806 |
